# State Medical Associations

**Published:** 1874-04

**Authors:** 


Transactions of the Medical Association of the State of Alabama; annual session of 1873, held in Tuscaloosa, March 25th, 26th and 27th. Selma, Ala., 1874. Pamphlet, pp. 112.

---

Transactions of the Indiana State Medical Society, 1873. Twenty-third Annual Session. Indianapolis. Pamphlet, pp. 145.

---

Transactions of the State Medical Society of Michigan, for the year 1873. Lansing, 1873. Pamphlet, pp. 170.

---

Transactions of the Maine Medical Association, 1873. Portland, Vol. IV, Part 3, 1873. Pamphlet, pp. 355 to 502.

---

Calendar of the University of Michigan, for 1872–'73. Ann Arbor. Published by the University, 1873. Pamphlet, pp. 88.

---

Second Biennial Report of the State Board of Health of California, for the years 1871–'72 and 1873. Sacramento: 1873. Pamphlet, pp. 235.

---

Valedictory Address—Delivered at the Annual Commencement of the College of Physicians and Surgeons, Baltimore, Maryland, by Professor E. Lloyd Howard, M.D. Quarto, pp. 3.

---

Inaugural Address of Clarke Bell, Esq., before the Medico-Legal Society of the city of New York, November 26, 1873. New York: 1874. Pamphlet, pp. 23.

